# Combining multi-marker metabarcoding and digital holography to describe eukaryotic plankton across the Newfoundland Shelf

**DOI:** 10.1038/s41598-022-17313-w

**Published:** 2022-07-29

**Authors:** Liam MacNeil, Dhwani K. Desai, Maycira Costa, Julie LaRoche

**Affiliations:** 1 Biology Department, Dalhousie University, 1355 Oxford St, Halifax, NS B3H 4J1 Canada; 2 Department of Biology and Pharmacology, Dalhousie University, 5850 College St, Halifax, NS B3H 4R2 Canada; 3 Department of Geography, University of Victoria, STN CSC, PO Box 1700, Victoria, BC V8W2Y2 Canada; 4 Present Address: GEOMAR Helmholtz Centre for Ocean Research Kiel, Düsternbrooker Weg 20, 24105 Kiel, Germany

**Keywords:** Computational biology and bioinformatics, Ecology, Molecular biology, Optics and photonics

## Abstract

The planktonic diversity throughout the oceans is vital to ecosystem functioning and linked to environmental change. Plankton monitoring tools have advanced considerably with high-throughput in-situ digital cameras and genomic sequencing, opening new challenges for high-frequency observations of community composition, structure, and species discovery. Here, we combine multi-marker metabarcoding based on nuclear 18S (V4) and plastidial 16S (V4–V5) rRNA gene amplicons with a digital in-line holographic microscope to provide a synoptic diversity survey of eukaryotic plankton along the Newfoundland Shelf (Canada) during the winter transition phase of the North Atlantic bloom phenomenon. Metabarcoding revealed a rich eukaryotic diversity unidentifiable in the imaging samples, confirming the presence of ecologically important saprophytic protists which were unclassifiable in matching images, and detecting important groups unobserved or taxonomically unresolved during similar sequencing campaigns in the Northwest Atlantic Ocean. In turn, imaging analysis provided quantitative observations of widely prevalent plankton from every trophic level. Despite contrasting plankton compositions portrayed by each sampling method, both capture broad spatial differences between the northern and southern sectors of the Newfoundland Shelf and suggest complementary estimations of important features in eukaryotic assemblages. Future tasks will involve standardizing digital imaging and metabarcoding for wider use and consistent, comparable ocean observations.

## Introduction

Eukaryotic plankton are enormously diverse and drift with surrounding water masses, also contributing to ecosystem function through primary and secondary productivity, mediating carbon sequestration along with other oceanic elements, and containing potentially harmful blooming species^1^. Plankton monitoring tools using conventional microscopy are steadily being replaced by a new generation of optics in digital imaging instruments, equipped with algorithms to extract, store, and classify major plankton groups mostly within nano-mesoplankton (2 µm to 20 mm) size ranges and bearing morphologically distinct features^2,3^. Imaging provides quantitative observations of taxonomic and functional diversity while also capturing species behaviour with unintrusive, in-flow sampling designs^4^. Together, the enhanced sampling capacity and stored digital information in image data have tremendous potential for quantitative plankton ecology, especially for morphologically diverse eukaryotes^5,6^.

Numerous imaging instruments and modes currently exist for plankton (e.g., see Table 1 in^1^). Among these, digital holographic imaging based on wavefront diffraction has grown across various disciplines, configured with or without optical lenses and supported by increasingly efficient numerical reconstruction algorithms applied to off-axis and in-line illumination paths for high-resolution amplitude and phase images^7,8^. The ordinary holographic principle to reconstruct the wavefront diffraction of an object from a coherent light source (i.e., a laser) can also be extended to record dimensions of color from incoherent light, including fluorescence^8^. Instrument designs without objective lenses or other opto-mechanical parts are well purposed to sample a wide range of plankton in-situ where sufficient image resolution and throughput are available^9–11^. These requirements lend strongly to digital in-line holographic microscopes, which rely on numerical reconstruction of a wavefront from a coherent light source without an objective lens, effectively increasing the depth of field that can be acceptably focused without sacrificing image resolution^12^. Using a single coherent light source, inflow designs allow in-line holographic microscopes an enhanced depth-of-field to image larger volumes for high-throughput sampling with simple designs for deployment, while simultaneously recording pixel intensity, amplitude, and phase shift at micrometer scales^13,14^. Lensless digital in-line holographic microscopes can produce comparable image resolution to high-end conventional light microscopy with orders of magnitude greater field-of-view^15^ and genus-level resolution is often achievable in plankton^16–18^. These traits make digital in-line holography especially applicable for sampling plankton and particulates in their environment^19^.

Any single imaging instrument has limited resolution and records a fraction of the plankton size spectrum, unlike DNA sequencing technologies which have revealed large portions of the oceans hidden, morphologically indistinct, and unculturable diversity^20^. The bulk pool of marine environmental DNA (eDNA) has recovered previously unexpected eukaryotic diversity^21,22^, global patterns of community structure^23^, and specific groups can be targeted using phylogenetic marker genes, known as metabarcoding^24^. Although no universal marker gene currently exists for hyperdiverse eukaryotic lineages, highly conserved markers include a nuclear target located on the small subunit of the 18S rRNA gene^25^, and a bacterial homologue to the cyanobacterial 16S rRNA gene present in the chloroplasts of photosynthetic protists^26,27^. Combining marker gene databases through multi-marker metabarcoding can reveal the often-unmanageable planktonic diversity present amongst the eukaryotes^28–31^. Due to the nature of the sequencing assay, sequence (read) counts do not reflect absolute abundances and must be treated as compositional^32^; reads are further decoupled from absolute abundances due to variable rRNA gene copy numbers, especially in the nuclear 18S rRNA gene, which can vary orders of magnitude in protists^33–35^ even among closely related groups^36^. Therefore, despite extensive taxonomically resolved compositions produced by metabarcoding, these methods do not yet recover quantitative assessments or morphological information (e.g., sex, life-stage, behaviour) available in microscopy.

**Figure 1:**
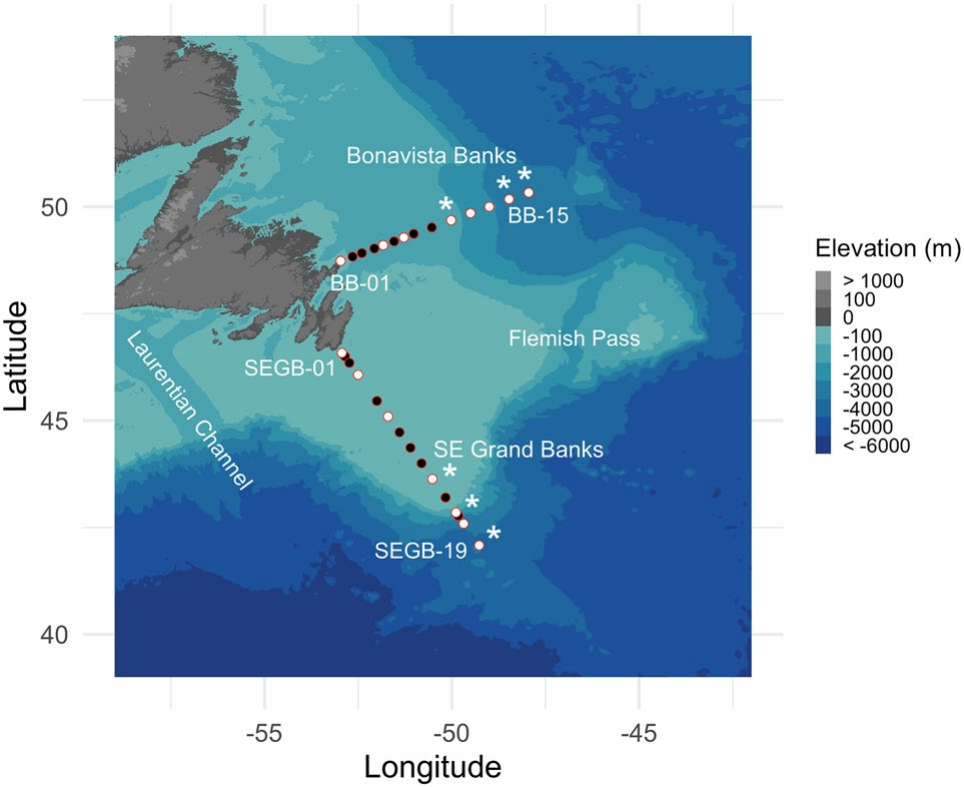
The Bonavista Banks (BB) and SE Grand Banks (SEGB) sampling transects. Stations (white and black) contain full CTD profiles, white dots indicate Niskin bottle samples for DNA and imaging at 5 m, and the asterisks indicate additional Niskin samples from 20 and 50 m. Note that the ship did not sample every original station, but original station names are kept here, thus SEGB-19 was the 15^th^ station sampled at the SE Grand Banks. Maps were generated using the *marmap* R package (v. 1.0.6)^82^.

Here we combine digital in-line holographic microscopy (HoloSea S5) and multi-marker metabarcoding (18S V4 and 16S V4-V5) resolved to single nucleotide Amplicon Sequence Variants (ASVs)^37^ with conductivity-temperature-depth (CTD) profiles including in-situ chlorophyll-a fluorescence to describe eukaryotic plankton composition > 10 µm in the Newfoundland Shelf (Canada) surface waters (Fig.1). This approach ensured identical volumes were imaged and sequenced, and together revealed a complex community of eukaryotic plankton supported by quantitative assessments of microplankton (> 20 µm) and mesoplankton (> 200 µm) from every trophic level. Plankton compositions displayed notable differences between transects and across the shelf-slope gradient. These results build on recent work characterizing seasonal planktonic diversity in the Northwest Atlantic Ocean, including observations of previously unobserved taxa that contribute significantly to spring blooms off-shelf, by providing taxonomically detailed compositions on this prominent coastal shelf during the 2019 early winter phase of the North Atlantic bloom phenomenon^38^.

## Results

### Multi-marker sequencing characterizes eukaryotic diversity

**Figure 2:**
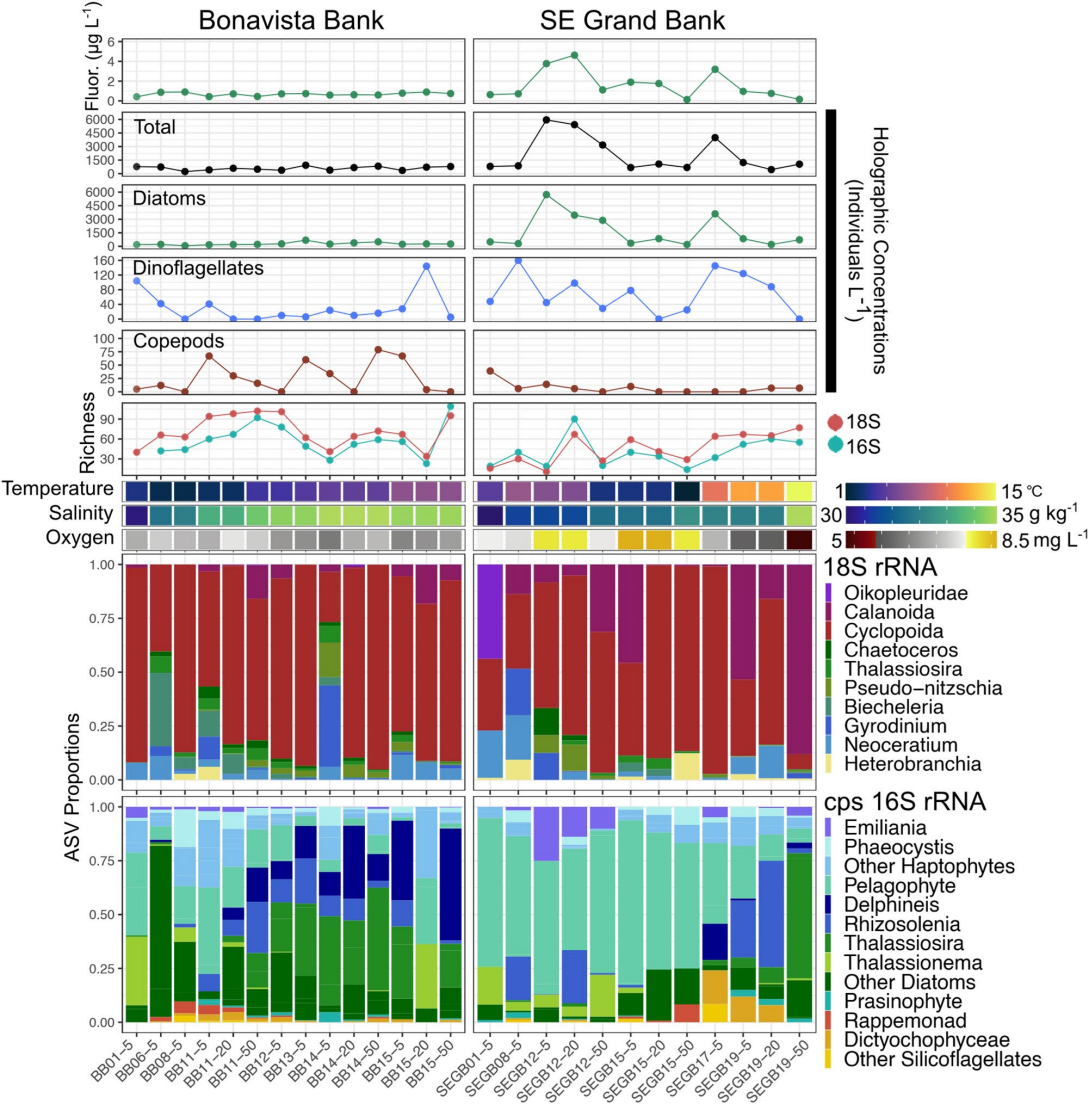
The full set of observations for both transects including chlorophyll-a fluorescence (Fluor.), imaging concentrations, ASV richness, physicochemical data (temperature, salinity, oxygen) and the multi-marker taxonomic composition. Richness error bars represent standard errors under the breakaway model. The x-axis is oriented by station order from shore to shelf and depth (5–50 m). Comparing transects reveals a contrast between higher plankton richness and lower abundance on Bonavista Banks and vice versa on the SE Grand Banks. Physical gradients spanning on-shelf to off-shelf water masses are also evident in salinity on the Bonavista Banks and temperature on the SE Grand Banks; however the 18S and 16S taxonomic compositions across these gradients are more complex during the early winter sampling period.

The combined set of CTD-derived physicochemical variables, quantitative imaging, and ASV richness with community composition from 18 and 16S rRNA genes is shown in Fig. 2. Combining 18S and 16S markers revealed rich eukaryotic compositions on the Newfoundland Shelf. After strict quality filtering (See Methods), 18S sequences (> 332 K) were classified to 237 ASVs and 16S sequences (> 99 K) were classified to 293 ASVs. A full list of unique genera classified from both markers is shown in Fig. S6. Heterotrophs dominated 18S rRNA sequence proportions owing to the copepod order Cyclopoida, which contributed up to 86% on the Bonavista Banks (Fig. 2) but was unclassified to lower taxonomic ranks. Four copepod species were classified in the order Calanoida (*Calocalanus curtus*, *Centropages hamatus*, *Paracalanus parvus*, and *Temora longicornis*) throughout our samples at low proportions (< 1%), although reaching > 80% of total sequence proportions at 50 m off shelf (SEGB-19) at the SE Grand Banks. Animals were also detected belonging to the gastropods (*Heterobranchia)* and Appendicularia (*Oikopleuridae*), constituting a notable fraction of ASVs (> 40%) to the SE Grand Banks coastal station (SEGB-01). A saprophyte genus of Labyrinthulomycetes (*Aplanochytrium*) was detected in rare proportions (< 1%) of 18S ASVs at off shelf stations on both Bonavista Banks and the SE Grand Banks. Phytoplankton usually composed < 30% of 18S ASVs mostly belonging to four dinoflagellate genera: *Tripos* (*Neoceratium*)*, Protoperidinium, Biechelaria,* and *Pelagodinium*. Diatoms contributed between < 1 and 10%, consisting of ASVs classified as *Chaetoceros*, *Guinardia*, *Pseudo-nitzchia*, and *Thalassiosira*.

Despite numerous phytoplankton classified in 18S ASVs including dinoflagellates, which were undetected in our chloroplast 16S V4–V5 targets and in other V1–V2 hypervariable regions^39^, the 16S show phytoplankton diversity is drastically underestimated (Fig. 2). Picophytoplankton classified as pelagophytes were widely distributed despite our 10 µm filter and were the main component (> 60%) on the SE Grand Banks. The diatoms were more diverse and contributed similar proportions on the Bonavista Banks, mostly belonging to *Thalassiosira*, *Thalassionema*, *Rhizosolenia* and *Delphineis*, among other rarer genera. Haptophytes contributed up to 25% of compositions as *Phaeocsystis* and *Emiliania*. Other rare phytoplankton > 10 µm were distributed across our samples including rappemonads and silicoflagellates mostly belonging to the family Dictyochophyceae.

**Figure 3:**
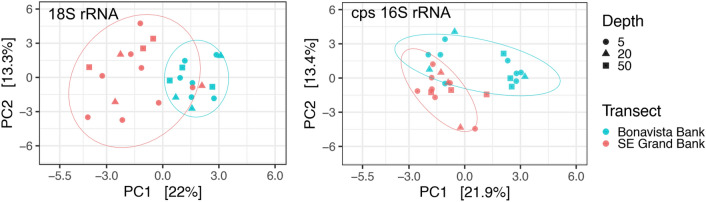
Principal Component Analysis (PCA) of 18S and chloroplast (cps) 16S rRNA markers showing the first (PC1) and second (PC2) most important components (axes) with shapes indicating sample depth (m). The ordination is calculated with an Aitchison’s distance^40^ a compositionally valid Euclidean distance of the clr-transformed ASVs.

ASV richness from 18S and 16S markers were generally in step, although 18S amplifications were richer overall (Fig. 2). Principal component analysis indicated an apparent clustering of samples within transects regardless of depth or amplicon marker (Fig. 3), indicating samples reflect surface water conditions only and were likely sampled above the mixed layer depth (Fig. S1). However, these results were not statistically significant for 18S (*p* = 0.165) or 16S (*p* = 0.114) using a permutational analysis of variance. Samples that co-clustered with opposing transects were characterized by relatively lower diversity as seen in two 18S compositions on the Bonavista Banks (BB-01, BB-15 at 20 m) dominated by arthropods (> 85%) (Fig. 2), and in several 16S samples where pelagophytes and diatoms contributed overwhelmingly (> 90%) to compositions. Distinct 16S compositions on the Bonavista Banks are found at the Northeast Slope on the Bonavista Banks where *Thalassiosira*,* Delphineis*, and *Rhizosolenia* and other diatoms contribute significantly to overall compositions.

### Imaging composition and concentrations

**Figure 4:**
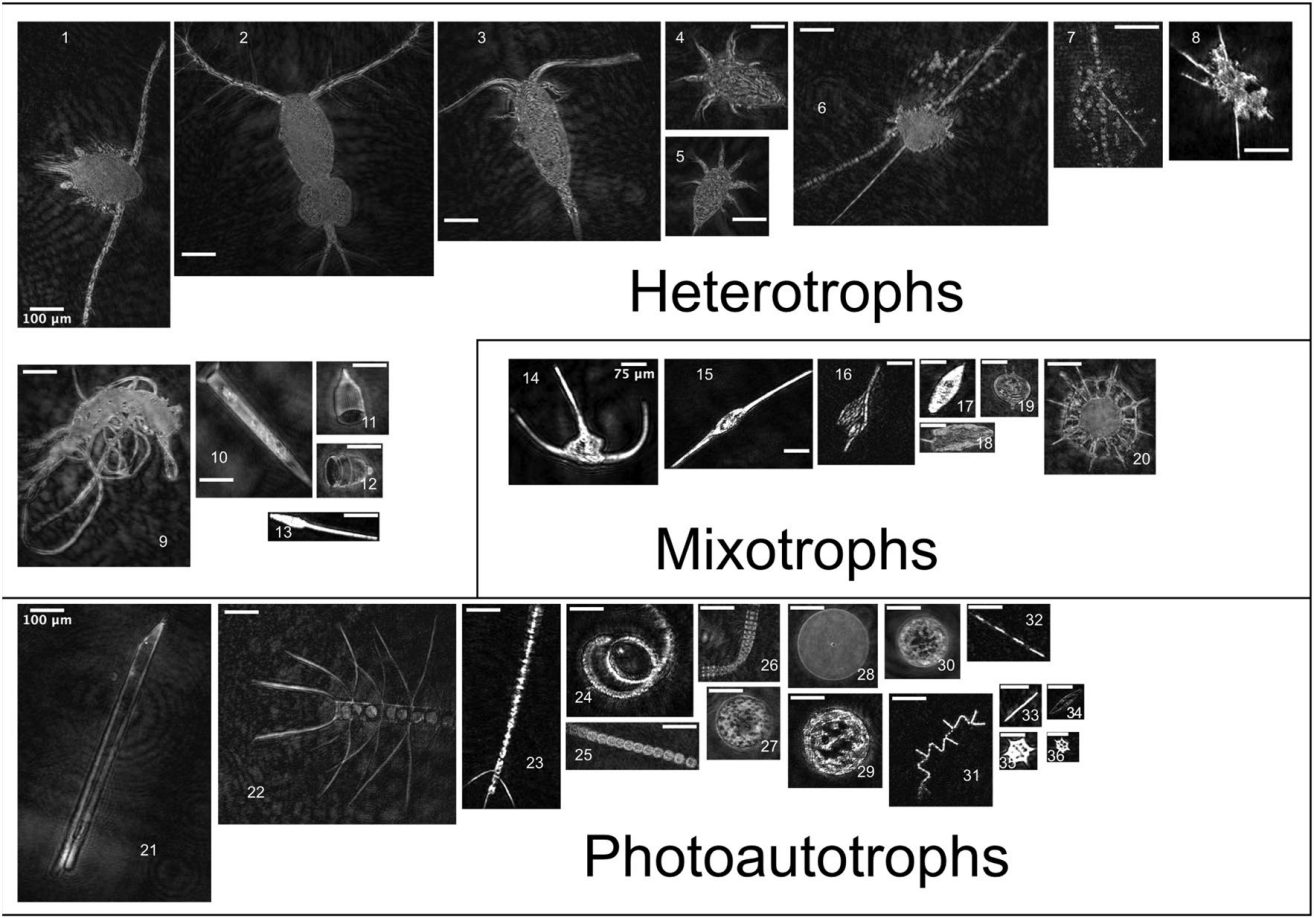
A collection of eukaryotic taxa observed on the Newfoundland Shelf. Groups are broadly divided into trophic level with respective scale bars, labelled at image 1, 14, and 21. Heterotrophs included adult copepods (1–3), larval nauplii (4–5), the saprophytic phylum Labyrinthulomycetes (6–8), the Amoebozoa Platyamoeba (9), tintinnids (10–12), and appendicularians (13). Mixotrophic dinoflagellates (14–19) included several genera of *Tripos* (14–16), with *Tripos fusus* (15) and *Tripos lineatum* (16), *Gyrodinium* (17), *Prorocentrum* (18), and *Protoperidinium* (19). The heterotrophic radiolarian Acantharia (20) commonly bears photosynthetic symbionts, creating mixotrophic nutrition^41^. The photosynthetic autotrophs included diatoms (21–35), and the silicoflagellate genera *Dictyocha* (35–36). The diatom genera included *Proboscia* (21), *Chaetoceros* (22–24), plus taxonomically unresolved chain-forming (25–26) and centric groups (27–30), *Thalassionema* (31), *Pseudo-nitzschia* (32), rod-shaped groups (33), and *Nitzschia* (34). To highlight additional data-driven benefits of holography, partially focused images (e.g., 21 and 23) produced from our methods can be refocused from raw holograms using, for example, an oblique reconstruction to recover obscured features outside the plane perpendicular to the optical axis^42^.

In total, > 55 K holograms were analyzed and > 105 K objects were detected. The bulk of objects were small (< 50 µm) non-living particles but > 4600 objects were taxonomically identified into 26 groups, ranging from phylum (e.g., Labyrinthulomycetes) to species-level (e.g., *Tripos fusus*), and large (> 50 µm) non-living particulates of marine snow and other aggregates were also counted. A collage is shown in Fig. 4. Imaged phytoplankton were overwhelmingly diatoms, dominated by chain-forming, centric, and rod-shaped cells. Three chained diatom genera (*Chaetoceros*, *Pseudo-nitzchia*, and *Thalassionema*) and two rod-shaped genera (*Proboscia* and *Nitzchia*) were identified, all of which were detected in the paired DNA samples, however most (> 80%) chained, centric, and rod-shaped diatoms lacked taxonomically diagnostic features and remained only morphologically identified. Dinoflagellates contained four physically defined genera (*Gyrodinium*, *Prorocentrum*, *Protoperidium*, and *Tripos*) but only *Tripos fusus* and *Tripos lineatum* were identified at a species level from holograms and furthermore only *Tripos fusus* was detected as *Tripos fusus f. tenuis* in matching DNA samples; all other *Tripos* (*Neoceratium*) groups were only classified to genus-level in 18S ASVs.

The heterotrophic taxa included larvacean zooplankton (Appendicularia), amoebozoans, copepods, tintinnids, and fungus-like protists. Among these, larvaceans and amoeba were broadly categorized, and copepods were differentiated by life stage (Fig. 4). The tintinnids contained at least two identifiable genera: *Salpingella* and *Condonella* from the phylum Ciliophora. The fungus-like protists belonged to the Labyrinthulomycetes phyla and were initially grouped with non-living particulates and only identified in images after DNA sequencing revealed their presence in specific samples. The Labyrinthulomycetes are increasingly recognized as ecologically important saprophytes and are described further in the discussion.

### Eukaryotic plankton across the Newfoundland Shelf gradient

The assemblages of eukaryotic plankton observed here in the early winter transition of the North Atlantic bloom phenomenon^38^ displayed notable differences between north and south sections on the Newfoundland Shelf. Temperature and Salinity (T–S) transitioned at both transects from relatively cooler, fresher shelf water towards warmer, saltier water masses off shelf (Fig. 2; Fig. S1). ASV richness was greater on the Bonavista Banks than the SE Grand Banks, highest towards the Northeast Slope (BB-11, BB-12) and at 50 m off shelf (BB-15) into the increasing gradient of temperature and salinity (Fig. 2, Fig. S1). These samples were characterized by generally low plankton concentrations observed in holographic images indicating smaller nanoplankton contribute to this diversity where microplankton concentrations captured in our images are low (Fig. 2). Conversely, the SE Grand Banks displayed rich phytoplankton samples near the shelf break (SEGB-12) seen in 16S ASVs coincident with high fluorescence (> 4 µg L^−1^) and concentrated diatoms (> 4500 individuals L^−1^) composed mostly of chains (Figs. 1, 3). Off-shelf of the SE Grand Banks (SEGB-17) a warm water mass contained a combined elevation of chlorophyll-a fluorescence with dinoflagellates (> 120 individuals L^−1^) and diatom (> 3000 individuals L^−1^) concentrations that steadily declined while richness increased towards the warmest off-shelf station (SEGB-19). Low autotrophic biomass on the Bonavista Banks was evident in both fluorescence and imaged plankton concentrations although no correlation was apparent; however imaged concentrations did co-vary with fluorescence on the SE Grand Banks (R^2^ = 0.76) (Fig. S7). Elevated plankton concentrations clustered on the shelf break of the SE Grand Banks containing centric (> 350 L^−1^), chain-forming (> 650 L^−1^) and rod-shaped (> 5000 L^−1^) diatoms, cooccurring with relatively high concentrations of the dinoflagellate genera *Tripos* (> 80 L^−1^).

## Discussion

The Newfoundland Shelf displayed a physical gradient transitioning across the continental shelf (Fig. 2; Fig. S3), capturing the temperate ocean water mass boundaries delimited by the SE Grand Banks and Bonavista Banks, with subpolar and subtropical water masses prevailing to the north and south, respectively^43^. This separation between shelf and open ocean waters is a ubiquitous feature of the Northwest Atlantic Ocean^44^ and can create biologically productive boundaries where (sub)mesoscale dynamics actively modify photosynthetically available radiation, nutrients, and predator encounter frequency^45^. The work described here combines high-throughput holographic imaging and multi-marker metabarcoding to characterize rich eukaryotic plankton communities (534 unique ASVs) across the Newfoundland Shelf gradient. By complementing imaging and multi-marker metabarcoding, broad quantitative and taxonomic differences were evident between north and south sections on the Newfoundland Shelf: Bonavista Banks contained low biomass, high richness samples characterized by relative higher proportions of diatoms and haptophytes; the SE Grand Banks revealed more complex eukaryotic compositions with notably high diatom concentrations near the continental margin, likely stimulated by upwelling of deep, cold, nutrient rich waters (> − 50°W; Fig. S1) that frequently characterize this region^46^. Taken together, complementing imaging and multi-marker metabarcoding afforded broad comparisons between plankton abundance, diversity, and taxonomic composition against broadly different environmental profiles.

Seasonal planktonic diversity has recently been characterized in surface waters between 37 and 66°N off the Newfoundland Shelf using chloroplast 16S (V1–V2) amplicon sequencing with conventional and imaging flow cytometry^39,47^ that provides important context to our findings. The off-shelf plankton compositions from the early winter transition phase (2015) were dominated by cyanobacteria and pico-phytoeukaryotes with large fractions of *Micromonas*, *Bathycoccus, Ostreococcus* (Clade I–II) and increasing proportions of pelagophytes northward^47^, for which we observe the opposite trend on the Newfoundland Shelf. The samples here omit most small eukaryotes due to > 10 µm filtration, although the pelagophyte picophytoplankton occurring throughout both transects were likely trapped in filter pores clogged by larger cells and in particle-associated forms, instead these results highlight that the numerically rare nano-microeukaryotes are highly diverse, especially diatoms, and patchy across the Newfoundland Shelf. We also recovered multiple dinoflagellate genera through 18S ASVs and imaging samples which were undetected with chloroplast 16S targets^39^ and taxonomically unresolvable from pigments^48^. Unravelling mixotrophic feeding strategies in dinoflagellates and other eukaryotic protists (e.g., radiolarians) to estimate feeding rates and biogeochemical fluxes will depend on adequate detection and behavioural observations^49^ that are possible with our combined holographic imaging and multi-marker metabarcoding approach. It is also observed that the important chain-forming diatom *Chaetoceros*, which rapidly contributes to phytoplankton compositions upon the initiation of the subpolar spring bloom, is undetected off-shelf in wintertime and is likely transported within water masses or experiences higher success during the winter-to-spring transition^47^; our observations suggest *Chaetoceros* and other springtime blooming plankton could be advected across the Newfoundland shelf-slope boundary, implying a possible pathway within the Labrador current export along the Flemish Cap and SE Grand Banks^50^.

The skewing of planktonic diversity from underlying absolute abundances in compositional sequencing data is due to several technical and biological factors. Principally, there are potentially large differences in sample sources between quantitative imaging and metabarcoding: Images collected here are mostly intact living cells, but both cellular and extracellular DNA exist in the bulk DNA pools of seawater, thus it cannot be determined what fraction of a sequenced sample is living metabolizing cells, dead cells, dormant cysts, or detritus^51,52^. For 18S compositions, arthropods likely dominated due to their multicellularity, although size fractioning through a larger pore size, e.g., > 250–500 µm as per conventional net tows^53^ would largely remove copepods to mitigate their overrepresentation during the amplification stage. Assigning ASVs as unique biological organisms is also limited by incomplete reference databases and biased for organisms of historical importance^54^. Work is still needed to understand biases in each marker gene target but extending to full-length gene sequencing platforms could superiorly resolve taxonomic composition and better account for intragenomic gene copies^55–57^. Altogether, this suggests that until quantitative community level sequencing is achieved^58^ it remains a separate, but complementary line of evidence with quantitative imaging towards plankton community composition.

Despite its limitations, metabarcoding benefitted the overall community assessment with a much deeper taxonomic resolution of the eukaryotes than paired images. Only 12 genera were identified across all imaging samples, whereas 78 unique genera were identified in the metabarcoding analysis. This resulted in important microbial eukaryotic groups undetected in our images but classified in ASVs. These include protists belonging to the major phyla Cercozoa in 18S ASVs, and haptophytes in the 16S ASVs from widely abundant coccolithophorid genera *Emiliania* and non-coccolithophorid genera *Phaeocystis*, which forms the densest colonial blooms in the North Atlantic and Southern Oceans^59^. Each group was likely unidentifiable in our holographic images due to their small cell size (< 50 µm) which has previously been difficult for recovering sharp images using the HoloSea^60^, and due to morphologically indistinct features. Metabarcoding also provided potential and validated taxonomic identities of unidentified image objects. Many detected rod-shaped and centric diatoms with distinct features remained taxonomically unresolved, however the corresponding ASVs suggest they likely belong to genera *Guinardia*, *Actinocylcus*, and *Thalassiosira*. The fungus-like Labyrinthulomycetes are the clearest example for improved taxonomy as they were missed during manual identification but classified as the genera *Aplanochytrium* in paired DNA samples; their absence in paired images directed a re-assessment of detected objects which led to 10 identified specimens. The Labyrinthulomycetes identified here belong to the order Labyrinthulida^61^, a dominant marine group also detected in the photic zone during the Tara Oceans expedition^22,61^—although the Tara expedition samples largely omit the Northwest Atlantic Ocean. A new ecological view of the Labyrinthulomycetes is growing with observations showing these protists can exceed prokaryotic biomass and frequently cooccur with fungi to saprophytically break down neutrally buoyant marine snow in the bathypelagic zone, significantly contributing to the metabolism of deep-sea ecosystems^62,63^.

Quantitative imaging by digital in-line holography captured abundant micro and mesoplankton from every trophic level using the pipeline for hologram reconstruction and object detection outlined in^60^. The HoloSea returned highly focused images for quantitative observations of major plankton clades in aquatic ecology (diatoms, dinoflagellates, ciliates, copepods, radiolarians, silicoflagellates, other large protists) but other important groups (coccolithophorids, haptophytes, other small flagellates, fish larvae) are currently omitted due to size, pixel resolution, or morphologically indistinct features. Future studies investigating plankton size structure from the HoloSea or similar holographic microscopes should implement a correction factor to account for the scattering of coherent light that biases size estimates non-linearly depending on object distance from the point source^64^. The promise for digital in-line holography is shared by digital imaging technologies more broadly, and is typically prohibitive for conventional light microscopy, which is to yield data-rich, high-frequency observations which to date have captured planktonic fish-larvae survivorship under river plume discharge regimes^65^, the evolution of particulate-mediated carbon export in rapidly changing marginal zones^66^, spatial patterns in plankton community compositions through time^67^, at regional scales^4,68^ and specific taxonomic lineages at global scales^69^. Novel biotic interactions have recently been observed using in-situ imaging, including pseudopodial feeding in acantharians from the East China Sea likely missed previously due to destructive sampling by conventional plankton nets^4^ and the frequent parasitization of the cosmopolitan copepod *Oithona* at the Scripps Pier in the Pacific Ocean^70^. Digital imaging can also capture size-structure patterns as an indicator of functional diversity^71^, which is a deeply evolutionary trait that affects ocean food webs and biogeochemistry^72,73^. The specialty of imagery for morphologically distinct eukaryotes is of particular importance because, unlike bacteria, morphology and behavior are central to eukaryotic diversity and ecology and metabarcoding is blind to these features^74^.

This work presents paired high-throughput imaging, metabarcoding, and physicochemical observations from the productive Newfoundland Shelf to show that the eukaryotic plankton fraction displays subregional variation across physical gradients, including patchy biogeography and biomass along the continental margin during the early winter bloom transition. As a geographic juncture between North Atlantic water masses and ongoing climate changes to the subarctic North Atlantic Gyre^75,76^, the Newfoundland-Labrador shelves deserve a more comprehensive study of planktonic diversity and variability using state-of-the-art molecular and optical tools to improve upon existing seasonal surveys relying on net tows, conventional microscopy, and bulk chlorophyll-a^77^ or flow cytometry^78^. The high-throughput, durable, and lensless designs for digital holographic microscopes supported by increasingly automated pipelines will make suitable complements with high-sensitivity molecular and oceanographic sensor platforms and promote further *in-situ* deployments to investigate plankton diversity and structure.

## Methods

### Study area

The Grand Banks of Newfoundland (Fig. 1) are a group of continental embankments where two major wind-driven Western Boundary Currents—the Artic-sourced Labrador Current and the North Atlantic Current—converge in the Northwest Atlantic Ocean^79^. The Grand Banks represent the foremost component of the Newfoundland-Labrador Shelves that supports one of the most seasonally productive regions in the Northwest Atlantic Ocean^80^. The data collected here is from two transects taken on the Newfoundland Shelf during the Atlantic Zone Monitoring Program (AZMP) 2019 cruise (*RRS James Cook; cruise JC190;* November 18–December 7, 2019). The Bonavista Banks transect contains 14 stations across > 400 km extending over the Northeast Slope subject to a majority of Arctic water influence. The Southeast (SE) Grand Banks transect includes 15 stations across > 550 km extending onto the Southeast Shoal, a uniquely shallow (< 90 m) ancient sandy plateau subject to intensive vertical mixing between the North Atlantic Current and the Labrador Current^81^.

### Oceanographic physicochemical data

Full water column profiles were obtained by lowering a conductivity-temperature-depth (CTD) rosette equipped with a calibrated fluorometer and a dissolved oxygen sensor (Seabird SBE-9+) from surface waters to 10 m above the seafloor. All CTD files were provided by Fisheries and Oceans Canada and handled in R using the *oce* package (v. 1.4.0)^83^. The CTD files were filtered in a standard fashion: CTD casts were trimmed to remove upcast anomalies, interpolated using the default method at 1 m increments, smoothed with local (Boxcar) averaging and gridded into sections^83^. The chlorophyll-a fluorescence (µg L^−1^) corresponding to the Niskin samples (Table S1) was extracted and averaged within three depth bins (5–6, 20–21, 50–51 m) to approximate conditions at Niskin bottle sample depths.

### Sample collection: paired imaging and filtration

Seawater was collected using 20 L Niskin bottles attached to the CTD rosette, emptied directly into 10 L cubitainers where a minimum of 2 L sample volume was allocated for paired imaging of micro and mesoplankton and filtration of eDNA. The full dataset includes 27 water samples from 15 stations (See Table S1) at 5 m, seven from the SE Grand Banks and eight from Bonavista Banks; three shelf break stations along each transect were also analyzed at 20 and 50 m. First, seawater was pumped at 150 mL min^−1^ using a peristaltic pump (Fisherbrand GP1000) into a digital in-line holographic microscope, the HoloSea S5 (Described below) at 10 frames s^−1^ and the seawater was collected in a container for further filtration for DNA extraction. The lower size limit of the object detection algorithm for the HoloSea S5 was set at 20 μm as a conventional lower size threshold to capture microplankton and larger objects^60^. Directly after imaging, samples were pumped through sterile tubing (Masterflex) and filtered onto 10 μm polycarbonate Isopore Membrane filters (Millipore, United States). Each filter was handled with ethanol-sterilized tweezers, promptly stored at -80 °C.

### Digital in-line holographic imaging

The HoloSea (92 × 351 mm, 2.6 kg) is a digital in-line holographic microscope using a solid-state laser (386 nm) as a point source emitted through a 0.5 µm pinhole located 54 mm distance from the monochrome complementary metal–oxide–semiconductor (CMOS) camera with 7.4 µm per pixel in the resultant 2048 × 2048 hologram. Further specifications and theoretical background for the HoloSea and 4-Deep software are described in^60^. To briefly detail the acquisition process of plankton objects, first raw holograms are reconstructed in 4-Deep Octopus software using a Helmholtz-Kirchhoff transformation and subsequently, using the 4-Deep Stingray software, biological Regions of Interest (ROIs) are detected using a global adaptive thresholding and filtered for in-focus objects in each reconstructed plane^60^. These in-focus objects compose our quantitative profiles which were manually identified to the lowest possible taxonomic rank (^84^Table S2). Concentrations for each sample were calculated by dividing the total number of individuals by the total volume that was imaged, calculated by multiplying the number holograms by the effective volume per hologram (0.063 mL) of the HoloSea^64^.

### DNA extraction

DNA was extracted from the 10 μm polycarbonate filters using the DNeasy Plant Mini Kit (Qiagen, Germany) according to the manufacturer’s instructions with modifications adapted to in-house reagents for the cell lysis procedure detailed in the Supplementary Material. The remaining steps to isolate and elute the DNA contents followed the manufacturer’s protocol. The final DNA elution included 100 μL of elution buffer. The DNA concentrations and purity were measured with a NanoDrop 2000 (Thermo Scientific, United States). The final DNA aliquot (27 μL) was stored at − 80 °C until further analysis.

### Library preparation and illumina MiSeq sequencing

PCR amplification, multiplex library preparation, quantification, and sequencing by Illimina MiSeq were performed at Integrated Microbiome Resources (IMR; Halifax, NS). Raw sequences can be found in under the NCBI Sequence Read Archive BioProject PRJNA803249. Samples were amplified using dual-indexing Illumina fusion primers targeting the V4 508 bp region in the 18S rRNA gene (E572F—CYGCGGTAATTCCAGCTC; E1009R—AYGGTATCTRATCRTCTTYG)^85^ and the V4–V5 371 bp region of chloroplast 16S rRNA gene (515FB—GTGYCAGCMGCCGCGGTAA; 926R—CCGYCAATTYMTTTRAGTTT)^86,87^. Data processing followed the Microbiome Amplicon Sequencing Workflow^88^ within the QIIME2 platform (v. 2020.8)^89^. Preprocessing sequence reads for quality control and generating stitched reads follow^88^ and are detailed in the Supplementary Material. To derive single nucleotide resolution between high-quality reads and determine ASVs^37^, Deblur^90^ was used to subtract erroneous reads based an upper-bound error profile from the sequence-to-sequence Hamming distances of neighbouring reads. During this step, we also positively filtered for 18S sequences using the PR2 (v. 4.14) 18S rRNA database^91^. The sequences were trimmed at 350 bp for 18S, and 360 bp for 16S sequences to remove low-quality reads^92^.

### Bioinformatic analysis

The ASVs were taxonomically classified using the multinomial naïve-Bayes QIIME2 q2-feature-classifier plugin^93^. The 18S sequence classifier was pre-trained in-house on the SILVA (v. 138.1) database^94^, and the chloroplast 16S sequences were classified in a two-step process: First sequences were run against SILVA (v. 138.1) database to extract chloroplast sequences to be classified from the PhytoREF database^27^. The classified ASVs were then filtered in two ways: (1) Rare ASVs were removed if they were less than 0.1% of mean sample depth in accordance with Illumina’s estimation of bleed-through errors during sequencing, (2) all ASVs only classified to the kingdom level were excluded. The remaining ASVs were exported from QIIME artifacts into R (v. 3.6.2)^95^ as a Phyloseq object (v. 1.3.6)^96^ for further analysis.

Richness (alpha diversity) was estimated using ASVs as a surrogate in the R package breakaway (v. 4.7.3)^97^. The breakaway model attempts to adjust richness estimates for unobserved taxa and debias samples from differing sequencing depths^98^. To assess differences in community composition between samples (beta diversity), all ASVs were transformed using a centered log-ratio ($clr$) of a composition $x=({x}_{1},\dots ,{x}_{i},\dots ,{x}_{D})$ defined as:

1 $$clr\left(x\right)= \left[ln\frac{{x}_{1}}{g(x)}, \dots , ln\frac{{x}_{i}}{g\left(x\right)},\dots , ln\frac{{x}_{D}}{g(x)}\right]$$

where each ASV ($x$) is divided by the geometric mean of all ASVs in a sample given by $g\left(x\right)=\sqrt[D]{{x}_{i}\dots {x}_{D}}$^99^. To prevent undefined log-ratios, a pseudo-count of one was added to each zero^100^. To summarize compositional differences between transects a principal component analysis of the Euclidean (Aitchison) distances^40^ was performed on log-transformed ASV counts^37^ and tested for statistical significance using a permutational analysis of variance^101^ in the vegan package (v. 2.5.7)^102^.

## Supplementary Information

Supplementary Information.

## Data Availability

18S and 16S sequences are available under the NCBI Sequence Read Archive BioProject PRJNA803249. Additional plankton objects which are representative of major taxa found in this study are available (10.6084/m9.figshare.20259756) and the imaging datasets generated during the current study are available from the corresponding author(s) upon reasonable request.
