# Publisher Correction: Combining multi-marker metabarcoding and digital holography to describe eukaryotic plankton across the Newfoundland Shelf

**DOI:** 10.1038/s41598-022-20894-1

**Published:** 2022-10-06

**Authors:** Liam MacNeil, Dhwani K. Desai, Maycira Costa, Julie LaRoche

**Affiliations:** 1 Biology Department, Dalhousie University, 1355 Oxford St, Halifax, NS B3H 4J1 Canada; 2 Department of Biology and Pharmacology, Dalhousie University, 5850 College St, Halifax, NS B3H 4R2 Canada; 3 Department of Geography, University of Victoria, STN CSC, PO Box 1700, Victoria, BC V8W2Y2 Canada; 4 Present Address: GEOMAR Helmholtz Centre for Ocean Research Kiel, Düsternbrooker Weg 20, 24105 Kiel, Germany

Correction to: *Scientific Reports* 10.1038/s41598-022-17313-w, published online 29 July 2022

The original version of this Article contained an error in the order of the Figures. Figures 1 and 2 were published as Figure 4 and 1. As a result, Figures 2 and 3 were renumbered to Figures 3 and 4.

In addition, Reference 102 was incorrectly given as Reference 84:

The correct Reference 102 is listed below:

Oksanen, J., et al. *vegan: Community Ecology Package.* R package version 2.5–7 (2020). https://CRAN.R-project.org/package=vegan  .

As a result, in the Methods section, under the subheading ‘Bioinformatic analysis’,

“To summarize compositional diferences between transects a principal component analysis of the Euclidean (Aitchison) distances40 was performed on log-transformed ASV counts37 and tested for statistical signifcance using a permutational analysis of variance102 in the vegan package (v. 2.5.7)^84^.”

now reads:

“To summarize compositional diferences between transects a principal component analysis of the Euclidean (Aitchison) distances40 was performed on log-transformed ASV counts37 and tested for statistical signifcance using a permutational analysis of variance102 in the vegan package (v. 2.5.7)^102^.”

The original Article has been corrected.

