# Supplementary material for: Combining multi-marker metabarcoding and digital holography to describe eukaryotic plankton across the Newfoundland Shelf
Source: Sci Rep. 2022 Jul 29;12:13078. doi: 10.1038/s41598-022-17313-w (PMC9338326; doi:10.1038/s41598-022-17313-w)
Supplement: Supplementary file 1 — Supplementary Information. [file 41598_2022_17313_MOESM1_ESM.docx]

**Supplementary Material**

**Combining multi-marker metabarcoding and digital holography to describe eukaryotic plankton across the Newfoundland Shelf**

Liam MacNeil^1*^(https://orcid.org/0000-0002-4125-5240), Dhwani K. Desai^1-2^(https://orcid.org/0000-0002-1447-5360), Maycira Costa^3^(https://orcid.org/0000-0002-1876-3351), Julie LaRoche^1^ (https://orcid.org/0000-0003-4809-6411)

^1^Biology Department, Dalhousie University, 1355 Oxford St, B3H 4J1, Halifax, NS, Canada,

^*^ Current address^:^ GEOMAR Helmholtz Centre for Ocean Research Kiel, Düsternbrooker Weg 20, 24105, Kiel, Germany

^2^Department of Biology and Pharmacology, Dalhousie University, 5850 College St, B3H 4R2, Halifax, NS, Canada

^3^Department of Geography, University of Victoria, PO Box 1700 STN CSC, V8W2Y2, Victoria, BC, Canada

Correspondence: [L.macneil@dal.ca](mailto:L.macneil@dal.ca) and [Julie.Laroche@dal.ca](mailto:Julie.Laroche@dal.ca)

Table S1. All 27 samples from 15 stations, ordered from on-shelf to off-shelf, on the SE Grand Banks (SEGB) and Bonavista Banks (BB) analyzed for discrete water samples, with complete coverage for metabarcoding and holographic imaging. The final dataset totals > 1 TB of image data and > 5 GB of sequencing data. The grey columns indicate the stations sampled at 5, 20, and 50 m, with volume sampled, total holograms, and estimated volume imaged per depth.

| Station | SEGB-01 | SEGB-05 | SEGB-08 | SEGB-12 | SEGB-15 | SEGB-17 | SEGB-19 | BB-01 | BB-06 | BB-08 | BB-11 | BB-12 | BB-13 | BB-14 | BB-15 |
| --- | --- | --- | --- | --- | --- | --- | --- | --- | --- | --- | --- | --- | --- | --- | --- |
| Lat (°N),  Lon (°W) | 46.58, -52.93 | 46.07,  -52.5 | 45.09,  -51.7 | 43.63,  -50.51 | 42.85,  -49.88 | 42.58,  -49.68 | 42.08,  -49.27 | 48.3  -52.96 | 49.1,  -51.83 | 49.28,  -51.28 | 49.68,  -50.01 | 49.85,  -49.5 | 50,  -49 | 50.17,  -48.47 | 50.33,  -47.95 |
| Time | Day | Day | Night | Night | Day | Night | Night | Day | Night | Night | Day | Night | Night | Night | Day |
| Depth (m) | 5 | 5 | 5 | 5  20  50 | 5  20  50 | 5 | 5  20  50 | 5 | 5 | 5 | 5  20  50 | 5 | 5 | 5  20  50 | 5  20  50 |
| Holograms  (5, 20, 50m) | 1701 | 1694 | 1884 | 1667  1946  2177 | 1866  1799  1895 | 2204 | 2146  3793  1585 | 2455 | 1893 | 2739 | 2326  2608  1896 | 3116 | 2623 | 1998  3024  2000 | 2293  2206  2953 |
| Volume (L)  (5, 20, 50m) | 2.3 | 2.2 | 2.6 | 2.1  2.0  2.2 | 2.2  2.0  2.2 | 2.4 | 2.4  2.4  2.1 | 2.6 | 2 | 2.7 | 2.3 2.5 2.1 | 2.7 | 2.7 | 2.2 2.7 2.7 | 2.7  2.6  2.0 |
| Volume Imaged (mL)  (5, 20, 50m) | 107 | 106 | 119 | 110  112  137 | 117  113  119 | 139 | 135  239  105 | 155 | 119 | 173 | 147  164  119 | 196 | 165 | 126  190  126 | 144  139  186 |

.

### Oceanographic Physicochemical Data

### Physicochemical measurements (temperature, salinity, oxygen, and fluorescence) in the upper 150 m are shown in Figure S1. No aberrant measurements were flagged in the CTD casts. The available CTD variables included physicochemical data for temperature (°C), absolute salinity (g kg^-1^), oxygen (mg L^-1^), and chlorophyll-a fluorescence (µg L^-1^). Since seawater density (S_a_; kg m^-3^) is a function of heat content and dissolved salt concentrations, *in-situ* seawater density calculations incorporated each stations latitude, longitude, temperature, and absolute salinity according to the Thermodynamic Equation of Seawater (TEOS-10) protocol [1]. The physical and biological properties differed markedly between Bonavista Banks and SE Grand Banks transects (Figure S1). The Bonavista Banks section is horizontally structured with cold, mixed, and dense surface waters and low ambient fluorescence < 1.25 µg L^-1^ (Figure S1 A). The surface waters become increasingly warm (> 4 °C) and saltier (> 34 g kg^-1^) towards the shelf break of the Northeast Slope (> -50 °W) (Figure S1 B). Conversely, the SE Grand Banks is both vertically and horizontally structured with a stratified water column on the shelf containing warmer, less saline, less dense surface waters and a localized bloom indicated by relatively higher fluorescence (> 4 µg L^-1^) at the shelf break (> -51 °W)— i.e., Southeast Shoal (Figure S1 C). The higher fluorescence corresponds with intrusions of colder, marginally saltier, but overall denser water masses (Figure S1 C-D), where upward transport is evident. Both transects had the highest dissolved oxygen content (> 8 mg L^-1^) along the coast above 50 m at the Bonavista Banks and below 50 m at the SE Grand Bank and. The SE Grand Bank slope also exhibits a highly oxygenated pocket co-incident with the productive shelf break.

### DNA Extraction and Library Preparation

### Extractions began by adding 50 of lysozyme (5 mg mL^-1^) (Fisher BioReagents, United Kingdom) to each filter, then vortexed on high for 30 s, followed by a five-minute incubation at room temperature. Then 400 μL of the DNeasy Plant Mini Kit lysis buffer AP1 was added to each filter plus 45 μL of proteinase K (20 mg mL^-1^) (Fisher BioReagents, United Kingdom). The samples were then incubated at 52 °C with shaking (300 rpm) for one hour. Once completed, 4 μL of RNase A (Qiagen, Germany) was added to the filters, vortexed and a 10-minute incubation— tubes were inverted twice during incubation to homogenize sample contents. Afterwards, according to the manufacturers protocol, 130 μL of DNeasy Plant Mini Kit Buffer P3 was added to the lysate, then incubated for five minutes on ice, centrifuged for five minutes (20,000 g), and the lysate was finally pipetted onto a spin column (Qiagen, Germany) to be centrifuged (20,000 g) for two minutes. The remaining steps to isolate and elute the DNA contents followed the manufacturer’s protocol. The final DNA aliquot (27 μL) was stored at -80 °C until further analysis.

PCR amplification, multiplex library preparation, quantification, and sequencing by Illimina MiSeq were performed at Integrated Microbiome Resources (Halifax, NS). Preprocessing of these sequences for bioinformatic analyses followed [2]: Paired-end sequencing reads were inspected for high-quality read pairs using FastQC (v. 0.11.8) [3], primers were removed using Cutadapt (v. 2.10) [4], forward and reverse reads were then stitched, and any low-quality or chimeric reads are filtered out using the default options [2] of VSEARCH (v. 2.7.0) [5].

### Bioinformatic Analysis

ASV richness (alpha diversity) was estimated across each transect in the R package breakaway (v.4.7.3) [6]. The breakaway model estimates unobserved taxa by fitting a non-linear regression to consecutive taxa frequency ratios (singletons, doubletons, tripletons, etc.) and predicts the number of unobserved taxa (i.e., The number of groups with a frequency of zero). Adjusting richness estimates for unobserved taxa and calculating model error debiases against differing sequencing depths across samples and allows more reliable comparison between samples [7]. Sequencing diversity was represented as phylogenetic trees (Figure S6) built for 16S sequences using a SATé-enabled phylogenetic placement (SEPP) of short amplicon fragments [8, 9] in a QIIME2 plugin. The 18S phylogenetic tree was built *de novo* by sequence alignment using Multiple Alignment using Fast Fourier Transform (MAFFT) [10], filtered for unconserved

and highly gapped regions [11], and a final tree was assembled and rooted at the midpoint of the largest tip-to-tip distance using FastTree [12].

### Plankton Community Composition and Diversity

The 27 samples from the Bonavista Banks and the SE Grand Banks produced, for the 18S rRNA sequencing, > 571K sequencing reads with 181 to 84,984 sequences per sample (median = 5846). Denoising retained > 337K sequencing reads ranging widely between 519 to 53,894 sequencing reads per sample (median = 3147). Removing shallow sequencing depths with 181 reads excluded SEGB-05 from subsequent analysis. Filtration for rare and kingdom-level classifications removed an additional 5557 reads and 1269 rare ASVs, resulting in a final > 332K sequencing reads belonging to 248 ASVs. The dataset contained 71 and 81% sparsity (zeros) in the Bonavista Banks and SE Grand Banks, respectively, with low singleton frequency (< 1%) for both transects. The cps 16S rRNA sequences contained > 2.03M reads with 494 to 486,003 reads per sample (median = 21,345). After denoising, > 1.37M reads were retained with 311 to 342,398 reads per sample (median = 14,560). Filtering for plastidial sequences against the PhytoREF database resulted in a final > 99 K sequencing reads belonging to 579 ASVs, with 1857 median reads per sample. The dataset contained 90 and 91% sparsity (zeros) in the Bonavista Banks and SE Grand Banks, respectively, with singleton frequency of < 7% on the Bonavista Banks and < 4% on the SE Grand Banks.

### Imaging Plankton Concentrations

For imaging concentrations, a linear regression compared estimated plankton concentrations with the binned fluorescence values for both transects, using a Model II regression for uncertainty associated with all measurements [13]. Taxonomic identification reference descriptions are given in Table S2.


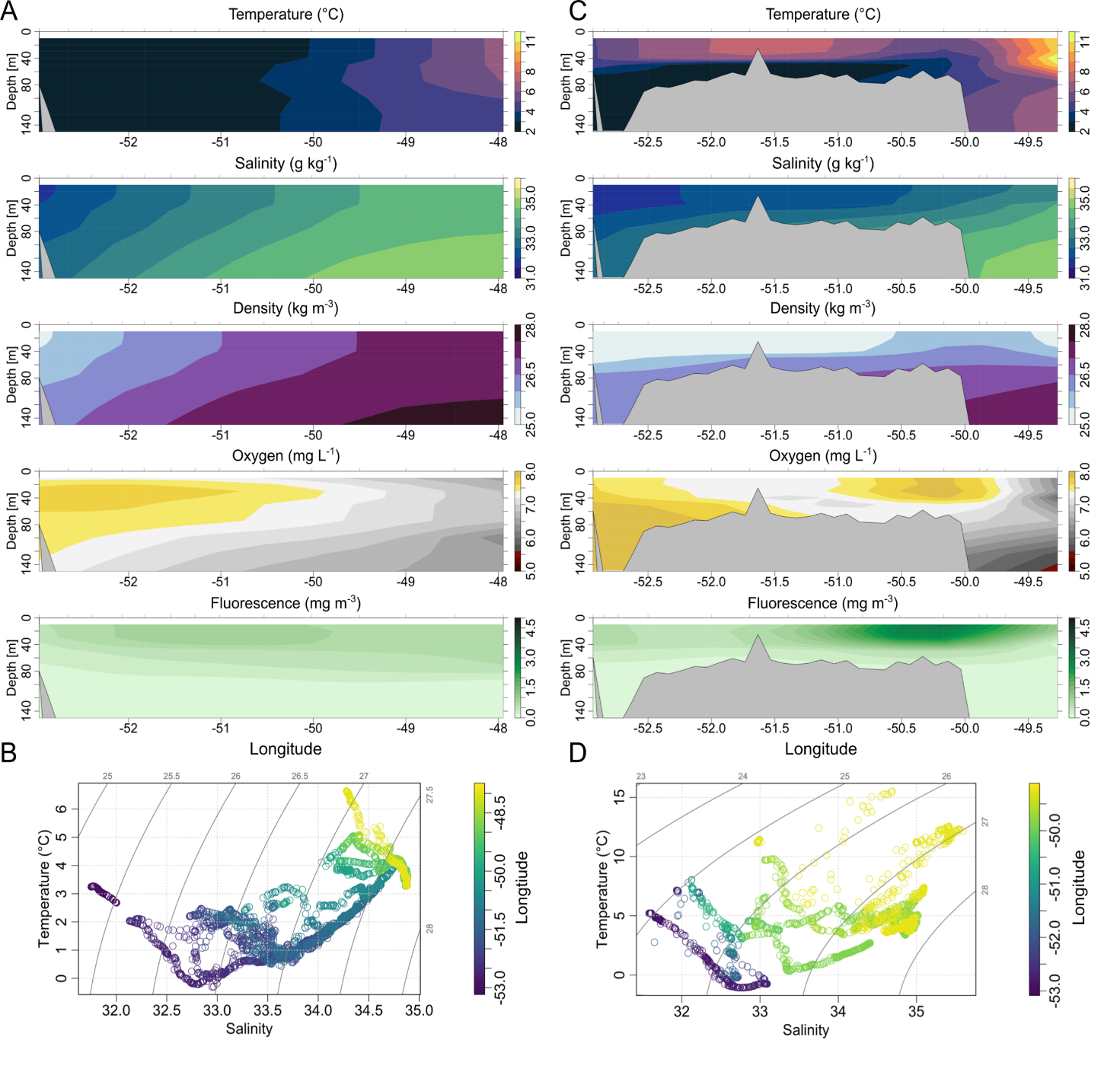


Figure S1. The 150 m section plots for A-B) Bonavista Banks and C-D) SE Grand Banks with corresponding temperature-salinity diagrams across the longitudinal gradient of the shelf. The location of each profile cast is indicated as tick marks on the top x-axis of each section plot. The isopycnals on the temperature-salinity diagrams indicate constant density. Section plot colormaps were taken from cmocean, which correct visual biases in classic oceanography jet colormaps that create arbitrary maximum and minimums within the color palette, regardless of the underlying data [14].


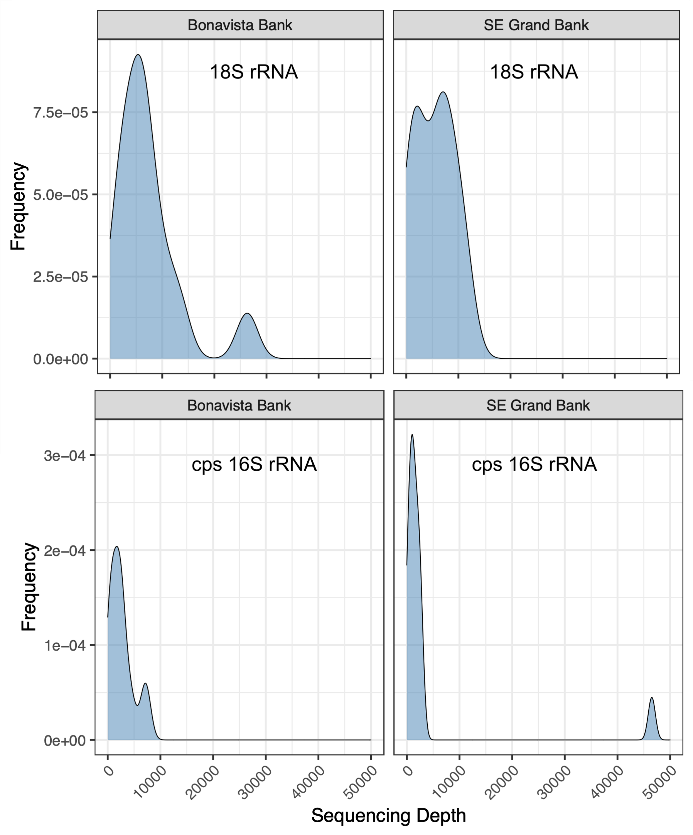


Figure S2. Sequencing depth by transect for the 18S rRNA and cps 16S rRNA datasets.

Figure S3. Scatterplots between genus-level richness and temperature for both 16S and 18S rRNA datasets.

Figure S4. Scatterplots between genus-level richness and oxygen for both 16S and 18S rRNA datasets.


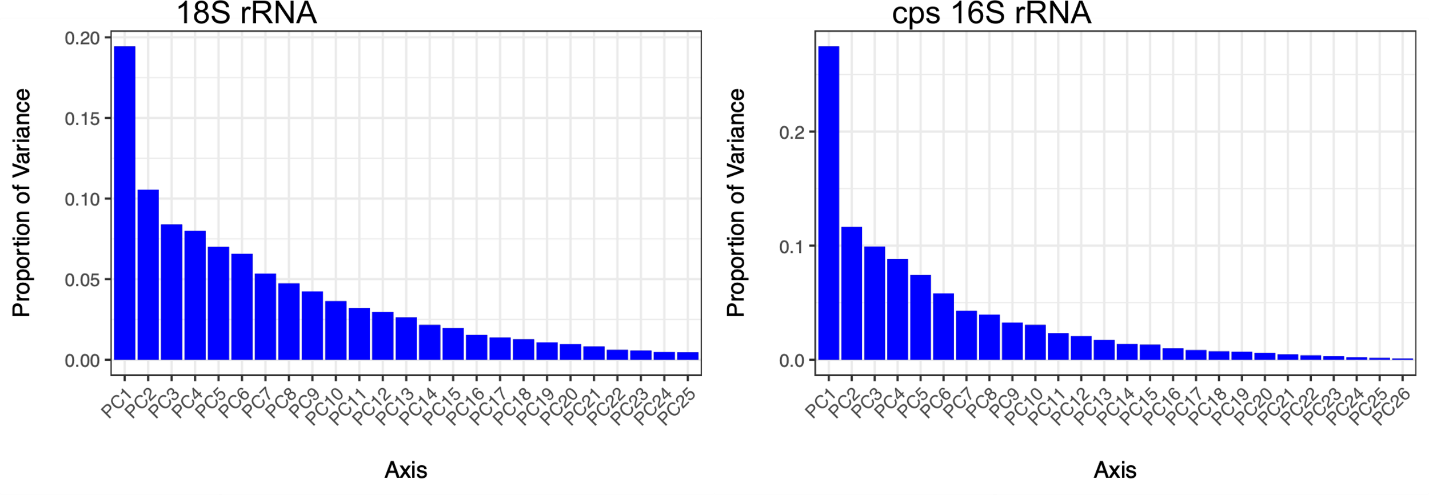


Figure S5. The variation explained by each axis in the principal component analysis (PCA) for 18S and cps 16S rRNA markers, respectively.


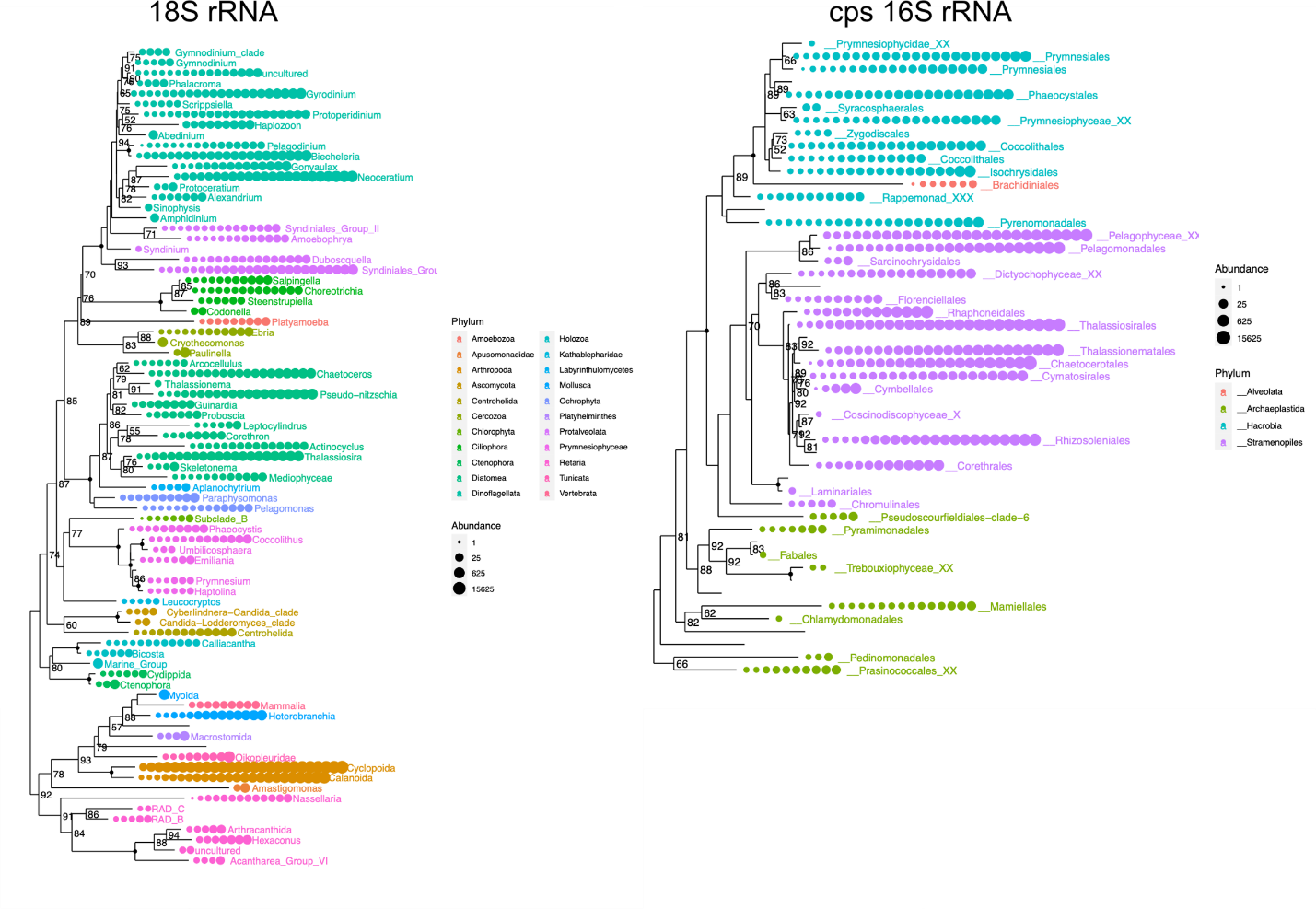
Figure S6. Phylogenetic trees for both 18S and cps 16S markers including sequencing read abundances at node tips colored by phylum and labelled by genus. The differences in phylum classification for identical groups (e.g., diatomea in 18S and stramenopiles in cps 16S) result from different annotations across the PR2 and PhytoREF databases. Confidence values are indicated by bootstrap estimates on each branch calculated using nodeplotboot in the plot_tree function of phyloseq (v.1.3.6).

Table S2. Description references for major plankton taxa including broad taxonomic group and genus where available. The Labyrinthulomycete genera was derived from corresponding 18S classification.

| Taxonomic Group | Genera | Description Reference |
| --- | --- | --- |
| Acantharia | NA | [15] |
| Copepods | NA | [16] |
| Ciliophora | *Codonella* | [17] |
|  | *Salpingella* | [17] |
| Centric diatom | *Chaetoceros* | [18] |
|  | *Proboscia* | [18] |
| Pennate diatom | *Pseudo-nitzschia* | [18] |
|  | *Nitzschia* | [18] |
| Diatom | *Thalassionema* | [18] |
| Dinoflagellate | *Tripos* (*Neoceratium*) | [19, 20] |
|  | *Tripos fusus* | [19, 20] |
|  | *Tripos lineatum* | [19, 20] |
|  | *Protoperidinium* | [19] |
|  | *Prorocentrum* | [19] |
|  | *Gyrodinium* | [19] |
| Labyrinthulomycetes | *Aplanochytrium* | [21] |
| Silicoflagellates | *Dictyocha* | [22] |


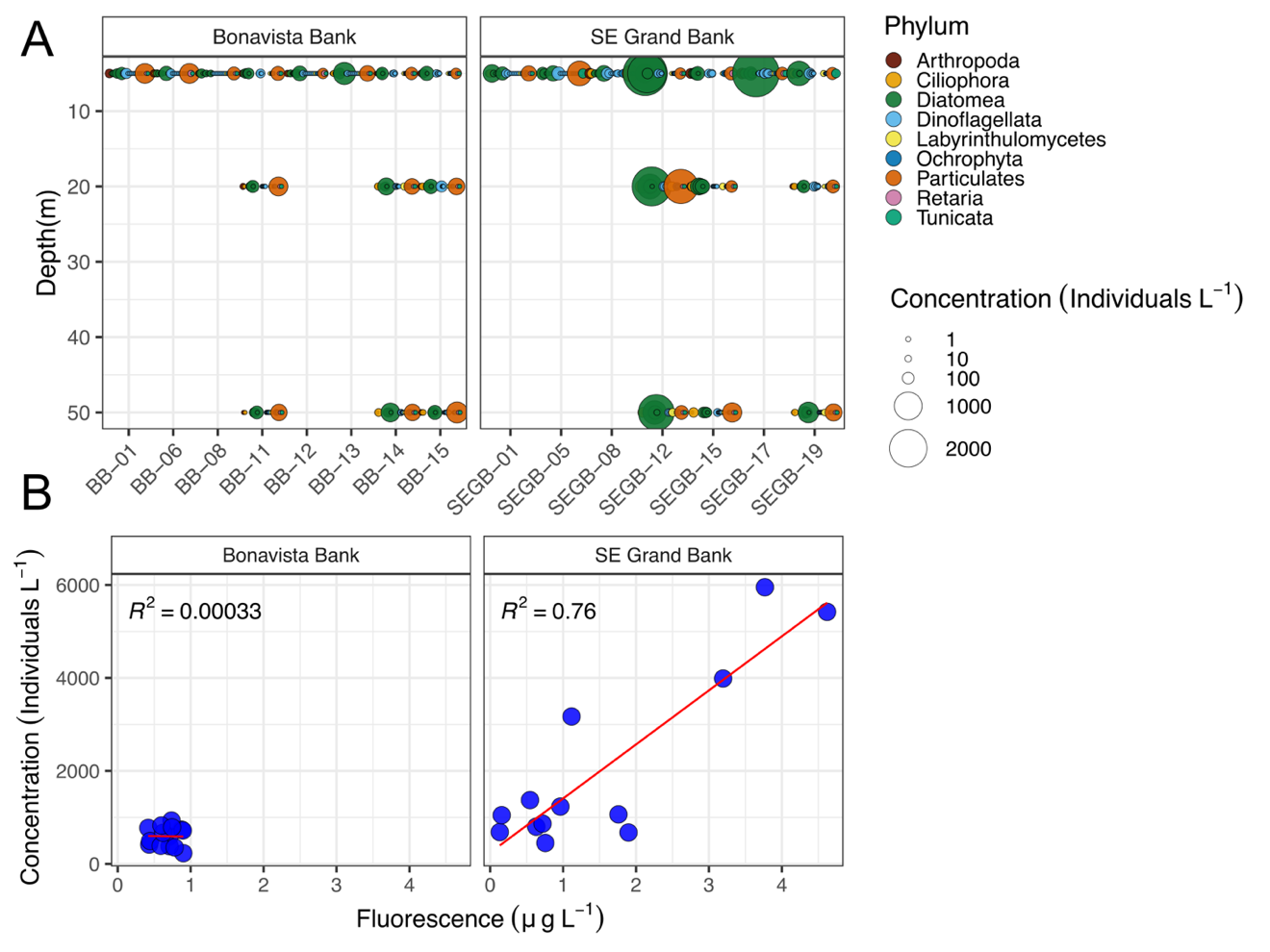


Figure S7. The imaging-based A) plankton concentrations oriented by longitude, from on to off-shelf stations. B) Correlation with the corresponding fluorescence measurements from both transects.

**References**

1. Wright, D. G., Pawlowicz, R., McDougall, T. J., Feistel, R., & Marion, G. M. Absolute Salinity, “‘Density Salinity’” and the Reference-Composition Salinity Scale: Present and future use in the seawater standard TEOS-10. *Ocean Science*, **7**(1), 1–26 (2011). <https://doi.org/10.5194/os-7-1-2011>.
2. Comeau, A. M., Douglas, G. M., & Langille, M. G. I. Microbiome Helper: A Custom and Streamlined Workflow for Microbiome Research. *MSystems*, **2**(1), *mSystems*.00127-16, e00127-16 (2017). <https://doi.org/10.1128/mSystems.00127-16>.
3. Andrews S. FastQC: a quality-control tool for high-throughput sequence data. Babraham Institute, Cambridge, United Kingdom. (2010): <http://www.bioinformatics.babraham.ac.uk/projects/fastqc/>.
4. Martin M. Cutadapt removes adapter sequences from high-throughput sequencing reads. *EMBnet. J.* **17** (2011).10_12 DOI 10.14806/ej.17.1.200.
5. Rognes T, Flouri T, Nichols B, Quince C, Mahe F. VSEARCH: a versatile open source tool for metagenomics *PeerJ*, **4,** e2584 (2016). <https://doi.org/10.7717/peerj.2584>.
6. Willis A, Bunge J. Estimating diversity via frequency ratios: Estimating Diversity via Ratios. *Biometrics*, **71**, 1042–1049 (2015). <https://doi.org/10.1111/biom.12332>.
7. Willis AD. Rarefaction, Alpha Diversity, and Statistics. *Front. Microbiol*. **10**, 2407 (2019). <https://doi.org/10.3389/fmicb.2019.02407>.
8. Mirarab, S., Nguyen, N., Warnow, T. (2011). SEPP: SATé-Enabled Phylogenetic Placement. *Biocomputing 2012*, 247–258. <https://doi.org/10.1142/9789814366496_0024>
9. Janssen, S., McDonald, D., Gonzalez, A., Navas-Molina, J. A., Jiang, L., Xu, Z. Z., Winker, K., Kado, D. M., Orwoll, E., Manary, M., Mirarab, S., Knight, R. (2018). Phylogenetic Placement of Exact Amplicon Sequences Improves Associations with Clinical Information. *MSystems*, **3**(3), e00021-18. <https://doi.org/10.1128/mSystems.00021-18>
10. Katoh, K., Standley, D. M. (2013). MAFFT Multiple Sequence Alignment Software Version 7: Improvements in Performance and Usability. *Mol. Biol. Evol.*, **30**(4), 772–780. <https://doi.org/10.1093/molbev/mst010>
11. Lane, D.J. (1991) 16s/23s rrna sequencing. In E Stackebrandt and M Goodfellow, editors, Nucleic Acid Techn. Bacterial System., pages 115–175. John Wiley and Sons, New York.
12. Price, M. N., Dehal, P. S., Arkin, A. P. (2010). FastTree 2 – Approximately Maximum-Likelihood Trees for Large Alignments. *PLoS ONE*, **5**(3), e9490. <https://doi.org/10.1371/journal.pone.0009490>
13. Laws, E. A., Archie, J. W. Appropriate use of regression analysis in marine biology. *Mar. Biol*. **65**, 13–16 (1981). doi: 10.1007/BF00397062
14. Thyng, K., Greene, C., Hetland, R., Zimmerle, H., & DiMarco, S. True Colors of Oceanography: Guidelines for Effective and Accurate Colormap Selection. *Oceanography*, **29**(3), 9–13 (2016). <https://doi.org/10.5670/oceanog.2016.66>.
15. Decelle, J., Not, F. Acantharia. In John Wiley & Sons Ltd (Ed.), *ELS,* pp. 1–10 (2015). <https://doi.org/10.1002/9780470015902.a0002102.pub2>.
16. Conway, D. V. P. Identification of the copepodite developmental stages of twenty-six North Atlantic copepods. Marine Biological Association of the United Kingdom. **21**, 28p (2006).
17. Dolan, J. R. The Biology and Ecology of Tintinnid Ciliates: Models for Marine Plankton, First Edition. Montagnes, D. J. S., Agatha, S., Coats, D. W., Stoecker, D. K. John Wiley & Sons, Ltd. Published (2013).
18. Hasle, G.R., Syvertsen, E.E. Marine Diatoms In: In: Tomas, C. R. (ed.) Identifying marine Phytoplankton. *Academic Press, Inc*., San Diego. 5-385 (1997).
19. Steidinger, K.A., Jangen, K. Dinoflagellates In: Tomas, C. R. (ed.) Identifying marine Phytoplankton. *Academic Press, Inc*., San Diego. 387-584 (1997).
20. Gómez, F. Reinstatement of the Dinoflagellate Genus *Tripos* to Replace *Neoceratium*, Marine Species of *Ceratium* (Dinophyceae, Aleolata). *CICIMAR Oceánides*, **28**(1), 1 (2013) <https://doi.org/10.37543/oceanides.v28i1.119>.
21. Leander, C. A., Porter, D., Leander, B. S. Comparative morphology and molecular phylogeny of aplanochytrids (Labyrinthulomycota). *European J. Protist.* **40**(4), 317–328 (2004). <https://doi.org/10.1016/j.ejop.2004.07.003>.
22. Throndsen, J. The planktonic marine flagellates. In: Tomas, C. R. (ed.) Identifying marine *Phytoplankton*. *Academic Press, Inc*., San Diego. 591-730 (1997).
